# A Rapid Colorimetric Method Reveals Fraudulent Substitutions in Sea Urchin Roe Marketed in Sardinia (Italy)

**DOI:** 10.3390/foods5030047

**Published:** 2016-06-25

**Authors:** Domenico Meloni, Antonio Spina, Gianluca Satta, Vittorio Chessa

**Affiliations:** 1 Department of Veterinary Medicine, University of Sassari, Via Vienna 2, 07100, Sassari, Italy; antospina@hotmail.it (A.S.); asd.areste@gmail.com (V.C.); 2 Dispatch centre “*Superfresco Luca*”, Agglom. Ind. San Marco, 07041, Alghero (SS), Italy; melonidome@hotmail.com

**Keywords:** sea urchin, roe, product replacement, egg yolk, colour

## Abstract

In recent years, besides the consumption of fresh sea urchin specimens, the demand of minimally-processed roe has grown considerably. This product has made frequent consumption in restaurants possible and frauds are becoming widespread with the partial replacement of sea urchin roe with surrogates that are similar in colour. One of the main factors that determines the quality of the roe is its colour and small differences in colour scale cannot be easily discerned by the consumers. In this study we have applied a rapid colorimetric method for reveal the fraudulent partial substitution of semi-solid sea urchin roe with liquid egg yolk. Objective assessment of whiteness (L*), redness (a*), yellowness (b*), hue (h*), and chroma (C*) was carried out with a digital spectrophotometer using the CIE L*a*b* colour measurement system. The colorimetric method highlighted statistically significant differences among sea urchin roe and liquid egg yolk that could be easily discerned quantitatively.

## 1. Introduction

The edible sea urchin (*Paracentrotus lividus*) is widely distributed in the Mediterranean Sea and along the North-eastern Atlantic coast [1,2] and is the most commercially exploited echinoid in Europe [3]. Sea urchin harvesting has been practised differently over the years among the geographical areas of the Mediterranean Basin, mainly in the southern regions [4,5,6,7,8]. In recent years, its populations have shown a wide scale decline in many European countries due to overfishing [1]. Sea urchin roe is a highly valued specialty food with a high-market demand in many Mediterranean regions [2,5,7,9,10] and in other non- Mediterranean European areas [11]. In Japan, sea urchin roe is often eaten as sushi, with demand for roe increasing as Japanese cuisine becomes popular in the North American food industry [12,13]. Due to the importance placed on product presentation in the sushi market, the quality of the roe is very important and influences the price for the product [14]. Market prices for sea urchin roe can range from US$6 to $200 per kilogram in the US [15] and from €75 to €100 per kilogram in Italy. The harvesting of *Paracentrotus* lividus is widespread in southern regions of Italy, [5]. In Sardinia (central-western Mediterranean), despite the fishery regulations (i.e., fishing periods, minimum size of harvestable individuals, and quantity per day per fisherman) [16] the harvesting of *Paracentrotus lividus* is intensively practised [2,17]. Previous surveys recorded that about 30 million sea urchins (1800 mt) are consumed every year, providing a turnover of more than €10 million. With 1.7 million inhabitants, Sardinia’s annual per capita consumption is about 1.1 kg—about four times the Japanese consumption [18], and its demand is constantly increasing [19]. Recent surveys highlighted the important role fulfilled by restaurants in the promotion of sea urchin roe in the dietary habits of the Sardinian population [20]. Sea urchin roe is widely used as a basic ingredient for several dishes (e.g., pasta, pizza, croutons). These dishes are characterized by high added value compared to that of basic ingredients, on which the restaurateurs count in order to build high profit margins for their whole activity [20]. These profit margins are so high as to more than counterbalance the risk of incurring penalties related to the fraudulent partial replacement of semi-solid sea urchin roe with surrogates that are similar in colour as liquid egg yolk, orangeade, and fruit juice. Small differences in colour cannot be easily discerned by the consumers. In the restaurant industry, where the complexity and the fragmentation of this sector prevent the implementation of continuous controls, these frauds are becoming widespread [21]. Sea urchin roe quality is strongly influenced by its appearance, colour, texture, and flavour [22]. In particular, the colour of the roe is one of the most important quality factors for its marketability [23,24]. It can range from a light yellow to a dark orange or almost red [12]. The yellowish-orange colour in the sea urchin roe is principally due to the pigment echinone [25], which is synthesised from beta-carotene by the sea urchin. The sea urchin obtains pigments through its diet [26]. Colour of the roe can be quantitatively measured by breaking it down into three components (L*a*b*) in a three-dimensional measurement such as the international standard developed by the Commission Internationele de l’Eclairage (CIE). The L*a*b* colour space is widely used in the food industry [27] and has been widely applied to study sea urchin roe [12,28]. The use of colorimetric methods to measure the colour of sea urchin roe in relation to habitat and nutrition has been widespread for over a decade [22,23,29,30,31]. However, to date, studies that focused on the use of colorimetric methods to assess the colour of marketed roe and reveal fraudulent substitutions have not been reported. In this study we have applied a rapid colorimetric method to reveal the most frequent fraudulent substitution of sea urchin roe with with surrogates that are similar in colour as liquid egg yolk, a product widely used in the restaurant industry. Our study was conducted on sea urchin roe obtained from *Paracentrotus lividus* populations harvested along the coast of Alghero (North-western side of Sardinia, Italy), where this typical activity plays a substantial socio-economic role [2]. It was hypothesized that a consumer could eat a dish with a mixture of semi-solid sea urchin roe and liquid egg yolk as ingredients because small differences in appearance and colour cannot be easily discerned. The colorimetric method should be a useful tool for the quantitative identification of differences among sea urchin roe and liquid egg yolk.

## 2. Materials and Methods 

### 2.1. Collection of the Samples

**Figure 1:**
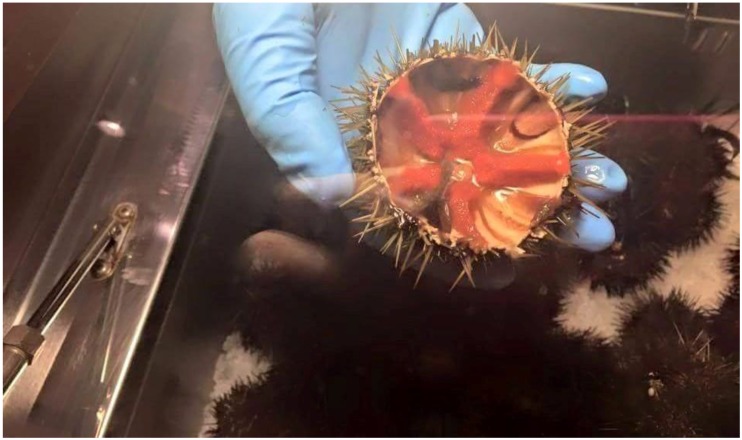
Opening of the sea urchin shells.

**Figure 2:**
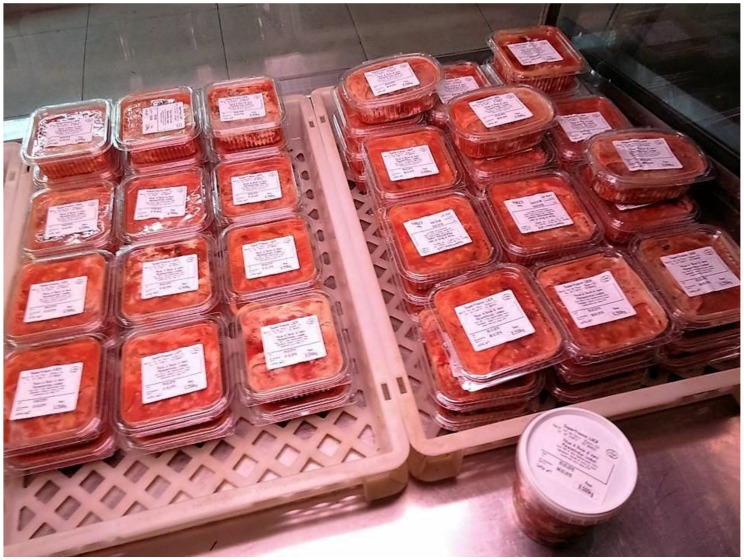
Sea urchin roe ready to be marketed.

Our study was conducted on sea urchin roe obtained from *Paracentrotus lividus* populations collected along the coast of Alghero (North-western side of Sardinia, Italy). From January to May 2015. Sea urchin specimens (>50 mm diameter) were hand harvested by professional fishermen in the Gulf of Alghero. Altogether, six different batches of sea urchin were included in our survey. Each batch, consisted approximately of 3000 specimens harvested daily. Live sea urchins were delivered to the local EU dispatch centre of Alghero for processing. The sea urchin shells were cracked open one at a time by hand, using a spade-like tool with sharp triangular blades (Figure 1). The blades break the shell in half, revealing the five undamaged roe. The roe was carefully removed using a tablespoon, cleaned of ingested food and adhering membranes using tweezers, and immediately placed in 500 g plastic containers. Approximately 150–200 specimens were required to obtain 500 g of sea urchin roe. Plastic containers were labelled and stored in a refrigerator room between 0 °C and +4 °C ready to be marketed (Figure 2). The shelf life declared by the producer on the label was five days. Two plastic containers of 500 g of sea urchin roe were randomly collected from each batch included in our survey. The 500 g package is the most widely used in restaurants. All of the items were identified with a univocal code and kept in ice boxes at +3 °C and were transported to the laboratory of the Department of Veterinary Medicine in Sassari and analysed on the same day.

### 2.2. Sensorial Analysis

A preliminary graded series of substitutions levels (90%, 80%, 70%, 60%, 50%) of semi-solid sea urchin roe with liquid egg yolk (purchased in a local shop) was used to define at what point the changes in appearance and colour were not detectable by a selected panel of five sea urchin roe consumers. Sensorial analysis has been carried out following the triangle test according to BS-ISO 4120:2004 [32]. During the triangle test, each panelist was presented with one different and two alike samples encoded anonymously with a three-digit code, presented in randomized order and balanced so as to be subjected in the first, second and third positions an equal number of times. This discriminative method allows identification of a perceptible sensory difference between two samples having small differences in sensory characteristics determined by several events; in this case, a change in composition. The sensory test sessions were performed in a room with artificial fluorescent lighting and set up in order to guarantee each panelist to carry out the test without mutual interference. The sensory sessions were held between 9:30 am and 11:00 am. The panel consisted of five untrained consumers aged between 20 and 45 years and classified as mere consumers with familiarity for the product. Each panelist was asked to identify the different sample with the forced choice method.

### 2.3. Preparation of the Samples for the Colorimetric Analysis

The samples were prepared according to the recommendations of the Commission Internationele de l’Eclairage [33] for the colorimetric analysis of semi-solid and liquid samples. The 500 g of semi-solid sea urchin roe were preliminarily homogenized and subdivided as follows. Ten “control” samples of sea urchin roe of 20 g each were obtained distributing 200 g of semi-solid sea urchin roe in 10 optical glass plates. Ten “experimental” samples of semi-solid sea urchin roe and liquid egg yolk (purchased in a local shop) as replacement were obtained by a preliminary homogenization of 100 g of sea urchin roe and 100 g of liquid egg yolk. The 200 g of experimental mixture (50% sea urchin roe and 50% liquid egg yolk) were distributed in 10 optical glass plates. Altogether, *n* = 120 samples (*n* = 60 “control” and *n* = 60 “experimental”) were subjected to colorimetric analysis.

### 2.4. Colorimetric Analysis

Colorimetric analysis for “control” and “experimental” samples was conducted using a digital Spectrophotometer Konica Minolta C508i (Konica Minolta Business Solutions Spa, Milan, Italy) using the CIE L*a*b* colour measurement system [33], standard illuminant D65, and 10° standard observer specular component included. The CIE L*a*b* formula defines colour breaking it down into three components (L*a*b*) in a three-dimensional measurement. The X axis, or redness (a*), extends from green on the negative side to red on the positive side. The Y axis, or yellowness (b*), extends from blue on the negative side to yellow on the positive. These two axes will define any colour. The intensity or whiteness of the colour is determined by the Z axis (L*), which extends from black on the negative side to white on the positive. Other properties, as hue (h*) and chroma (C*), may be computed together with by converting these coordinates from rectangular form to polar form. Hue is the angular component of the polar representation, while chroma is the radial component. The numerical values derived from this technique provide a true objective measure of the variance within a sample and, therefore, can be statistically evaluated. Each “control” and “experimental” sample was measured in triplicate and the averaged value was used in statistical analysis.

### 2.5. Statistical Analysis

Preliminary evaluation of homoscedasticity has been carried out prior to analysis of variance [34]. One-way ANOVA (α = 0.05) using the GLM procedure was performed to test the differences in whiteness (L*), redness (a*), yellowness (b*), hue (h*) and chroma (C*) between “control” and “experimental” samples. Statgraphics plus 5.1 (Statistical Graphics Corp., Rockville, MD, USA) was used for statistical analysis.

## 3. Results

**Figure 3:**
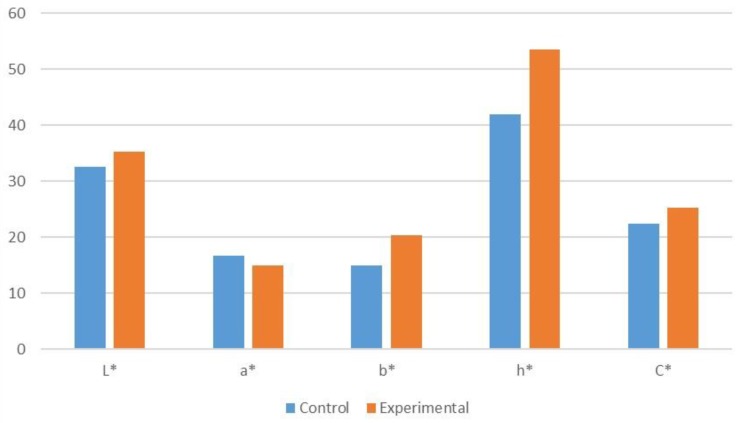
Colorimetric analysis of L*, a*, b*, C*, and h* parameters in “control” and “experimental” samples of sea urchin roe.

Substitutions of semi-solid sea urchin roe with liquid egg yolk at levels of 90%, 80%, 70%, and up to 60%, provided great differences in appearance and colour that were easily pointed out by the majority of the selected panel of sea urchin roe consumers. On the contrary, it was shown that a fraudulent partial substitution of up to 50% provides only small differences in appearance, flavour, and colour and these could not be easily discerned by the selected panel of sea urchin roe consumers. The objective assessment of whiteness (L*), redness (a*), yellowness (b*), hue (h*), and chroma (C*) in “control” samples using the CIE L*a*b* colour measurement system revealed an averaged number (±SD) of colour spaces as follows: L* = 32.56 ± 2.86; a* = 16.77 ± 1.65; b* = 15.04 ± 2.00; h* = 41.94 ± 1.53; C* = 22.53 ± 2.56, whereas for “experimental” samples, the following values were obtained: L* = 35.26 ± 1.81; a* = 14.93 ± 0.82; b* = 20.39 ± 2.48; h* = 53.50 ± 2.35; C* = 25.31 ± 2.46 (Figure 3). Colour-space parameters a*, b*, and h* differed significantly by one-way ANOVA (*p* < 0.05) between “control” and “experimental” samples (Table 1). 

## 4. Discussion

By using the CIE L*a*b* colour measurement system we have eliminated the subjective aspects of colour measurement [35]. The colorimetric method highlighted differences among sea urchin roe and surrogates that are similar in colour that could be easily discerned quantitatively. Statistical analysis reveals significant differences between “control” (100% sea urchin roe) and “experimental” (50% sea urchin roe and 50% liquid egg yolk) samples in the colour-space parameters a*, b*, and h*. The small differences of tone within a single colour displayed by sea urchin roe depends on individual subjectivity and can be a source of misinterpretation [31]. They cannot be easily discerned by the consumers and, at the same time, guarantee high profit margins to fraudulent restaurateurs. Colour measurements using digital instrumentation enabled us to detect such subtle differentiation in colour.

## 5. Conclusions

In the complex and fragmented restaurant industry, the main problems are related to the EU framework in terms of traceability [36], and to the use of surrogates that are similar in colour as liquid egg yolk generally accepted as being one of the most common food allergens [37]. According to Council Regulations (EC) 1169/2011 [38], allergen information for non-prepacked food, including in restaurants, are mandatory. Although the results of the present study must be considered preliminary, this is the first study focusing on fraudulent substitutions in sea urchin roe marketed in Sardinia (Italy). The rapid colorimetric method should be a practical and useful tool for the quantitative identification of differences among sea urchin roe and liquid egg yolk. To better understand the mechanism of fraudulent substitution of sea urchin roe in the restaurant industry, further testing with other surrogates that are similar in colour and lower substitution levels are needed in order to set at what point the differences in colour are detectable with colorimetric analysis. At this stage of marketing, a fraudulent use of 10%–40% of surrogates that are similar in colour would not be easily detected by the consumers, in terms of colour, and still increase the profit margins, leading to increased profit margins for fraudulent restaurants.

## Figures and Tables

**Table 1:** Results of one-way ANOVA for the effect of fraudulent substitution in the colour spaces of sea urchin roe L*, a*, b*, C*, and h*.

Source	*df*	L*			a*			b*			C*			h*		
		MS	*F*	*p*	MS	*F*	*p*	MS	*F*	*p*	MS	*F*	*p*	MS	*F*	*p*
*Between groups*	1	21.933	3.822	>0.05	10.146	5.944	<0.05	85.922	16.839	<0.05	23.192	3.669	>0.05	400.769	101.289	<0.05
*Within groups*	10	5.739			1.707			5.102			6.322			3.957		
*Total*	11															
